# Development and Validation of a Pyroptosis-Related Signature for Predicting Prognosis in Hepatocellular Carcinoma

**DOI:** 10.3389/fgene.2022.801419

**Published:** 2022-01-24

**Authors:** Jianfeng Ding, Xiaobo He, Wei Luo, Weiguo Zhou, Rui Chen, Guodong Cao, Bo Chen, Maoming Xiong

**Affiliations:** ^1^ Department of General Surgery, The First Affiliated Hospital of Anhui Medical University, Hefei, China; ^2^ Department of Radiology, The First Affiliated Hospital of Anhui Medical University, Hefei, China; ^3^ Department of General Surgery, Chaohu Hospital of Anhui Medical University, Chaohu, China

**Keywords:** pyroptosis, hepatocellular carcinoma, prognosis, tumor microenvironment, risk model, chemoresistance

## Abstract

Hepatocellular carcinoma (HCC) has emerged as a primary health problem and threat to global mortality, especially in China. Since pyroptosis as a new field for HCC prognosis is not well studied, it is important to open a specific prognostic model. In this study, consensus clustering method for 42 pyroptosis-related genes to classify 374 HCC patients in the TCGA database. After cox regression analysis of the differentially expressed genes between the two clusters, LASSO-Cox analysis was then performed to construct a pyroptosis-related prognostic model with 11 genes including MMP1, KPNA2, LPCAT1, NEIL3, CDCA8, SLC2A1, PSRC1, CBX2, HAVCR1, G6PD, MEX3A. The ICGC dataset was served as the validation cohort. Patients in the high-risk group had significantly lower overall survival (OS) rates than those in the low-risk group (*p* < 0.05). COX regression analysis showed that our model could be used as an independent prognostic factor to predict prognosis of patients and was significantly correlated with clinicopathological characteristics. Nomogram showing the stability of the model predicting the 1, 3, 5 year survival probability of patients. In addition, based on the risk model, ssGSEA analysis revealed significant differences in the level of immune cell infiltration and activation of immune-related functional pathways between high and low-risk groups, and patients with the high-risk score may benefit more from treatment with immune checkpoint inhibitors. Furthermore, patients in the high-risk group were more tend to develop chemoresistance. Overall, we identified a novel pyroptosis-related risk signature for prognosis prediction in HCC patients and revealed the overall immune response intensity of the tumor microenvironment. All these findings make the pyroptosis signature shed light upon a latent therapeutic strategy aimed at the treatment and prevention of cancers.

## Introduction

Hepatocellular carcinoma (HCC), accounting for approximately 90% of all primary liver cancers, is one of the most common and lethal malignancies in the world (Bray et al., 2018). Since the symptoms and physiological features of HCC are not easily detected at an early stage, making it usually impossible for 80% of patients to be treated by surgery at the time of diagnosis, the 5 year survival rate of their patients is still less than 20% despite great progress in current treatment strategies for HCC (Zongyi and Xiaowu, 2020).

Pyroptosis is a novel programmed cell death triggered by inflammatory bodies, which is characterized by the continuous expansion of cells until cell membrane rupture, resulting in the release of cellular contents and then causing a strong inflammatory response (Zhang et al., 2018; Frank and Vince, 2019). The occurrence of pyroptosis depends on the inflammatory caspase and GSDMs protein family. Simply put, the activated caspase cleaves the GSDMs protein and releases its N-terminal domain, which binds membrane lipids and punches holes in the cell membrane, resulting in changes in cell osmotic pressure, and then swells until the cell membrane ruptures (Ding et al., 2016; Feng et al., 2018).

The mechanism and function of pyroptosis in the tumor have been extensively studied, but its relationship with tumor prognosis is not clear. This is because of the complex interaction between pyroptosis and cancer, which leads to pyroptosis as an inflammatory death that can not only inhibit the progression of cancer but also promote tumor growth by providing a suitable microenvironment for tumor cells (Xia et al., 2019). Increasing studies have demonstrated that pyroptosis can promote immune evasion of tumor cells by disturbing the immune microenvironment. Luan et al. (Luan and Ju, 2018) described that activated caspase-1 stimulate pyroptosis and release pro-inflammatory cytokines, which exert a role in promoting HCC. Additionally, NLRP3 can induce pyroptosis and produce mature IL- 1β or IL- 18 to impair the host immune response in gastric cancer (Pachathundikandi et al., 2020). Therefore, further investigation of the role of pyroptosis in HCC is needed to provide new targets and biomarkers for individual treatment and prognosis of HCC.

Classification of HCC patients by high-throughput sequencing technology are a new method, which can accurately identify cancer features and guide clinicians in appropriate treatment strategies. It is therefore of outstanding interest to develop a brand-new gene signature associated with pyroptosis to evaluate the prognosis of individuals with HCC, especially the guidance of targeted therapy.

In the present study, we clustered 374 patients with HCC according to pyroptosis-related genes. On this basis, lasso-cox regression analysis was used to establish a pyroptosis-related risk signature, which represents an interesting new way to explore the prognostic value of patients with HCC, reflecting the immune microenvironment of the tumor and sensitivity to chemotherapy.

## Materials and Methods

### Data Acquisition

RNA sequencing data and corresponding clinical information of 374 HCC patients were downloaded from the TCGA database (http://cancergenome.nih.gov/) as a train set. Similarly, 231 HCC patients were obtained from ICGC (LIRI-JP) (https://dcc.icgc.org/) as a validation set. Patients with no survival information will be excluded from the cohort.

### Differentially Expressed Pyroptosis-Related Genes

We extracted 52 pyroptosis-related genes from previously published literature for the follow-up study, as shown in Supplemental Table S1. The “limma” algorithm in R software was performed to obtain the differentially expressed genes (DEGs) according to the screening criteria (*p*-value<0.05).

The STRING database (https://string-db.org/) was used to build a protein-protein interaction network (PPI) on DEGs and R software was carried out to analyze the inter-regulatory relationships between DEGs (cutoff = 0.4).

### Consensus Clustering

Consensus Clustering was performed to confirm different pyroptosis-related subtypes associated with pyroptosis regulators expression via the k-means clustering. The appropriate number of stable HCC clusters was calculated using a clustering algorithm in the “ConsensusClusterPlus” package. 1,000 iterations were performed to ensure the accuracy of the final classification. We screened the DEGs for subsequent analysis based on the samples in the different classifications obtained from the previous clustering analysis. |log2FC| >1 and adjusted *p*-value <0.05 were considered statistically significant.

### Construction and Validation of the Pyroptosis-Related Prognostic Signature

First, DEGs were subjected to univariate cox analysis to obtain genes associated with prognosis in patients with HCC (*p*-value<0.00001). The obtained prognosis-related genes were then used for Lasso-Cox analysis using the “glmnet” package with 10-fold cross-validation to prevent overfitting of the model, thus obtaining the genes and their coefficients for model construction. The formula for the risk score is as follows:

$$\text{Risk}\;\text{Score}=\sum_{i=1}^{N}\left(Exp_{i}\times Coe_{i}\right)\;$$

Where N = 11, *Exp*_*i*_ indicates the expression value of eleven genes, and *Coe*_*i*_ represents the coefficient of the corresponding gene. Patients were classified into high-risk and low-risk groups based on the calculated median risk score, and overall survival (OS) of patients with HCC between two groups was performed by Kaplan-Meier analysis using the “survival” and “survminer” packages. PCA analysis reduces the dimensionality of multivariate data to two or three principal components, which can be visualized graphically with minimal information loss. PCA analysis based on 11 genes signature was carried out by the “Rtsne” package. Univariate cox analysis was carried out to discern latent prognostic factors, and risk score determined by multivariate cox analysis could be used as independent prognostic factor for HCC patients. The ability of the risk model to predict prognosis in HCC patients was assessed using receiver operating characteristic curves (ROC) generated by the “SurvivalROC” package.

### Construction of a Prognostic Nomogram

We created a predictive nomogram based on risk score and clinicopathological characteristics to predict the OS probability of patients with HCC at 1, 3, and 5 years. Calibration plots were used to verify the accuracy of the prediction performance of the prognostic nomogram.

### Genetic Alterations and Functional Analyses

The Liver Hepatocellular Carcinoma (TCGA, Firehose Legacy) dataset which contained 379 patients were selected for alteration analysis of 11 genes from the cBioPortal (www.cbioportal.org). mRNA expression z-scores (RNA Seq V2 RSEM) were obtained using a z-score threshold of ± 2.0. Gene Ontology (GO) and Kyoto Encyclopedia of Genes and Genomes (KEGG) analysis based on DEGs were performed by employing “clusterProfiler” package. GSEA was conducted to examine a marked difference in the gene set between the low- and high-risk groups in the enrichment of the MSigDB cluster (c2. cp.kegg.v7.4. symbols.gmt). In addition, the activation of various immune cells subsets and immune-related pathways in high- and low-risk groups was examined by utilizing single-sample gene set enrichment analysis (ssGSEA). Expression levels of 47 immune checkpoints were evaluated in high and low-risk groups.

### Drug Sensitivity Assessment

The sensitivity of patients with HCC in high- and low-risk groups to four common chemotherapy agents was assessed via the Genomics of Drug Sensitivity in *Cancer* database (https://www.cancerrxgene.org/). Half maximal inhibitory concentration (IC50) was calculated by using “pRRophetic” package.

### Statistical Analysis

All strategy analysis is processed through R software (version 4.0.5). The categorical variables were analyzed using pearson’s chi-square test. Kaplan-Meier analysis and log-rank test were conducted to evaluate the statistical significance in OS between patients in high- and low-risk groups. Univariate and multivariate Cox regression analyses were applied to assess independent prognostic factors. Mann-Whitney test was used to evaluate the ssGSEA score for immune cell infiltration and immune pathway activation in the two risk groups.

## Results

### Identification of Pyroptosis-Related DEGs in Normal and HCC Tissues

**FIGURE 1:**
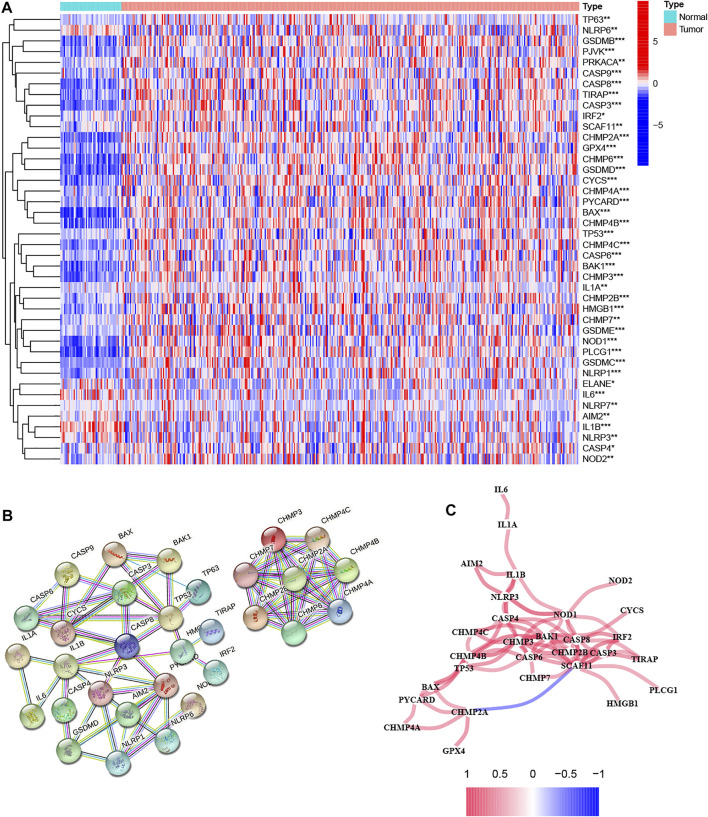
Analysis of the differential expression of pyroptosis-related genes in tumor and normal tissues and their inter-regulatory effects. **(A)** The heatmap showed the expression levels of 42 pyroptosis-related genes in tumor and normal tissues, where red indicates high expression and blue indicates low expression. *p < 0.05, **p < 0.01, and ***p < 0.001. **(B)** Protein–Protein Interaction interactions among hub pyroptosis-related DEGs. **(C)** The correlation network among pyroptosis-related DEGs, where red indicates positive regulation and blue indicates negative regulation. A darker color of the line between genes indicates a more significant correlation.

We first extracted 52 pyroptosis-related genes from the TCGA database and then performed differential expression analysis on them in normal and tumor tissues. The results of the heatmap demonstrated that 42 pyroptosis-related genes were identified as DEGs, of which 3 pyroptosis-related genes were downregulated in the tumor, while the remaining 39 genes were upregulated (Figure 1A). To better understand the mode of interaction between these pyroptosis-related DEGs, protein-protein interaction (PPI) analysis of DEGs was conducted with the highest confidence score (0.9) using Homo sapiens dataset, and PPI network retained 31 hub DEGs with complex regulatory relationships (Figure 1B). Furthermore, we calculated the correlation coefficients between genes based on the screening criterion (cutoff >0.4), and the results showed that most of these DEGs were positively regulated, except for CHMP2A and SCAF11, which were negatively regulated (Figure 1C). We preliminarily conclude that most of these pyroptosis-related differentially expressed hub genes affect tumor development and progression by means of positive regulation between each other.

### Identification of HCC Classification Based on 42 Pyroptosis-Related DEGs

**FIGURE 2:**
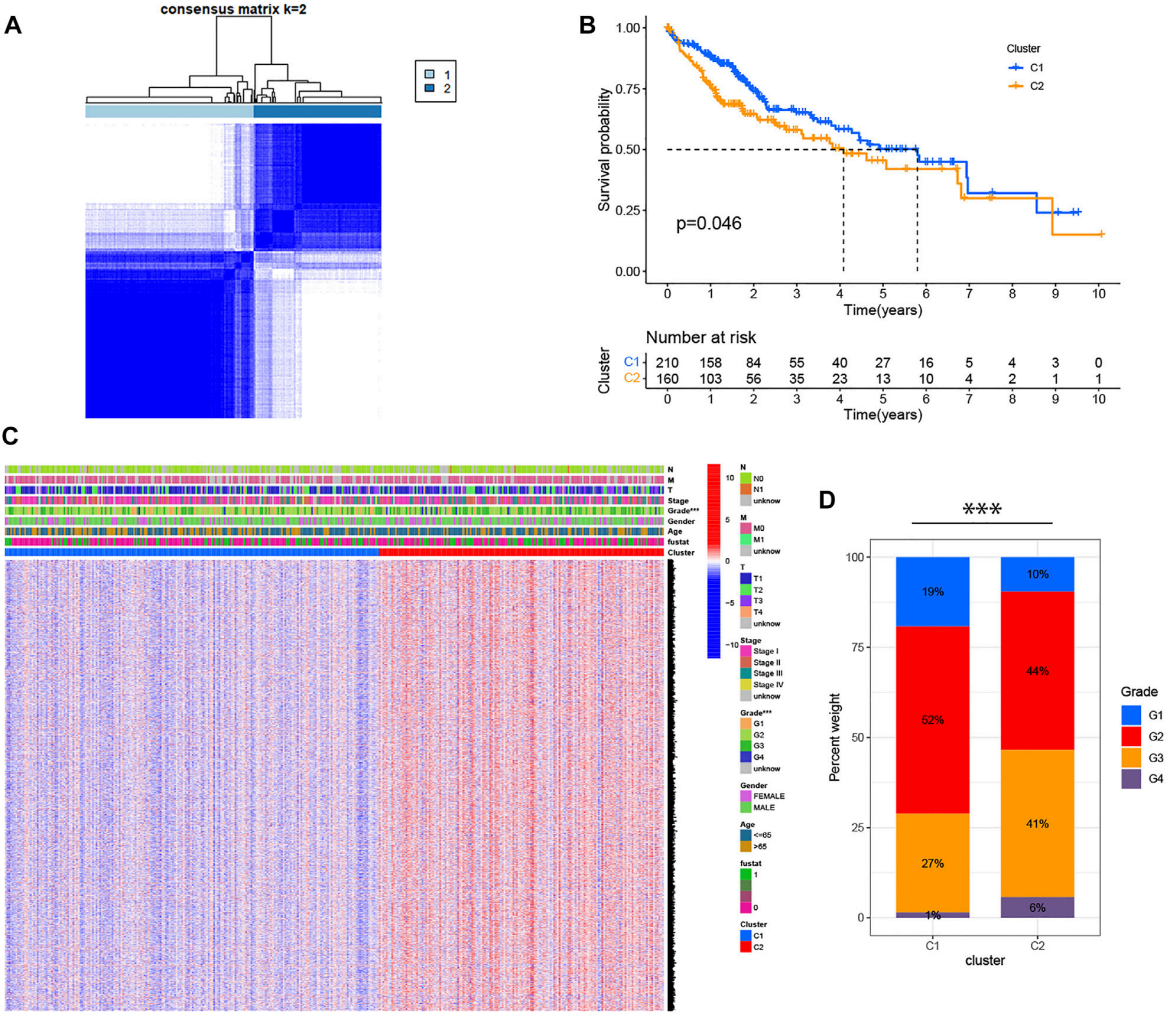
Identification of HCC classification based on pyroptosis-related DEGs. **(A)** 370 patients with HCC were divided into two clusters by the consensus clustering matrix (K = 2). **(B)** Kaplan-Meier OS analysis of HCC patients in two clusters. **(C)** Heatmap displaying the expression of DEGs in classification and the relationship between clinicopathologic characters and classification. “Fustat” represents the survival status, where “0” indicates that the patient is still alive and “1” indicates that the patient has died. ***p < 0.001. **(D)** The number of patients with different clinicopathological grades in two clusters.

Based on the expression of 42 pyroptosis-related DEGs together with patient survival information, we identified 2 clusters with unsupervised clustering methods in the TCGA cohort, containing 210 samples in cluster 1 and 160 samples in cluster 2 (Figure 2A). The result of the survival analysis demonstrated that the OS time of patients in cluster two was significantly poorer than that of cluster 1 (Figure 2B). To further explore the differences between the two clusters, we first screened and obtained the 2,291 DEGs of the two clusters according to the screening criteria (logFC>1, fdr<0.05). DEGs expression profiles and clinicopathologic characters comprising age, grade, stage, gender, and survival status were presented on the heatmap, and we found that the expression of most DEGs and the number of patients with high clinicopathological grade were significantly higher in cluster 2 (Figures 2C,D).

### GO and KEGG Analyses

**FIGURE 3:**
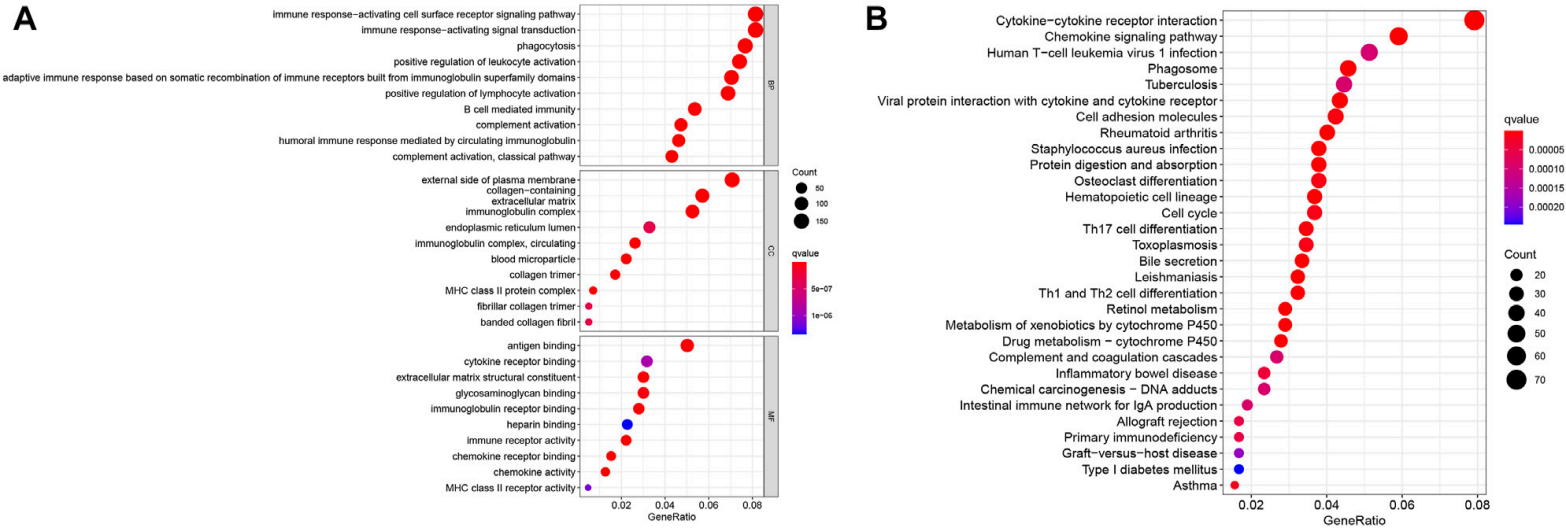
Enrichment analysis of the differentially expressed genes. **(A)** GO analysis. **(B)** KEGG analysis.

To further determine the potential function of DEGs between the two clusters, GO and KEGG analysis were conducted in R software. GO analysis was grouped into three parts: biological process (BP), cellular component (CC) and molecular function (MF). As displayed in Figure 3A, the results of the GO analysis revealed that these DEGs were abundantly enriched in various important immune responses, such as complement activation, B cell-mediated immunity, positive regulation of lymphocyte activation, humoral immune response mediated by circulating immunoglobulin, phagocytosis, immune response-activating signal transduction and immune receptor activity. Further, KEGG enrichment highlighted the role of Cytokine-cytokine receptor interaction, Chemokine signaling pathway, Cell cycle, Cell cycle, Th1 and Th2 cell differentiation, Drug metabolism, Primary immunodeficiency pathways, and so on (Figure 3B).

### Development of a Pyroptosis Risk Signature in TCGA Cohort

**FIGURE 4:**
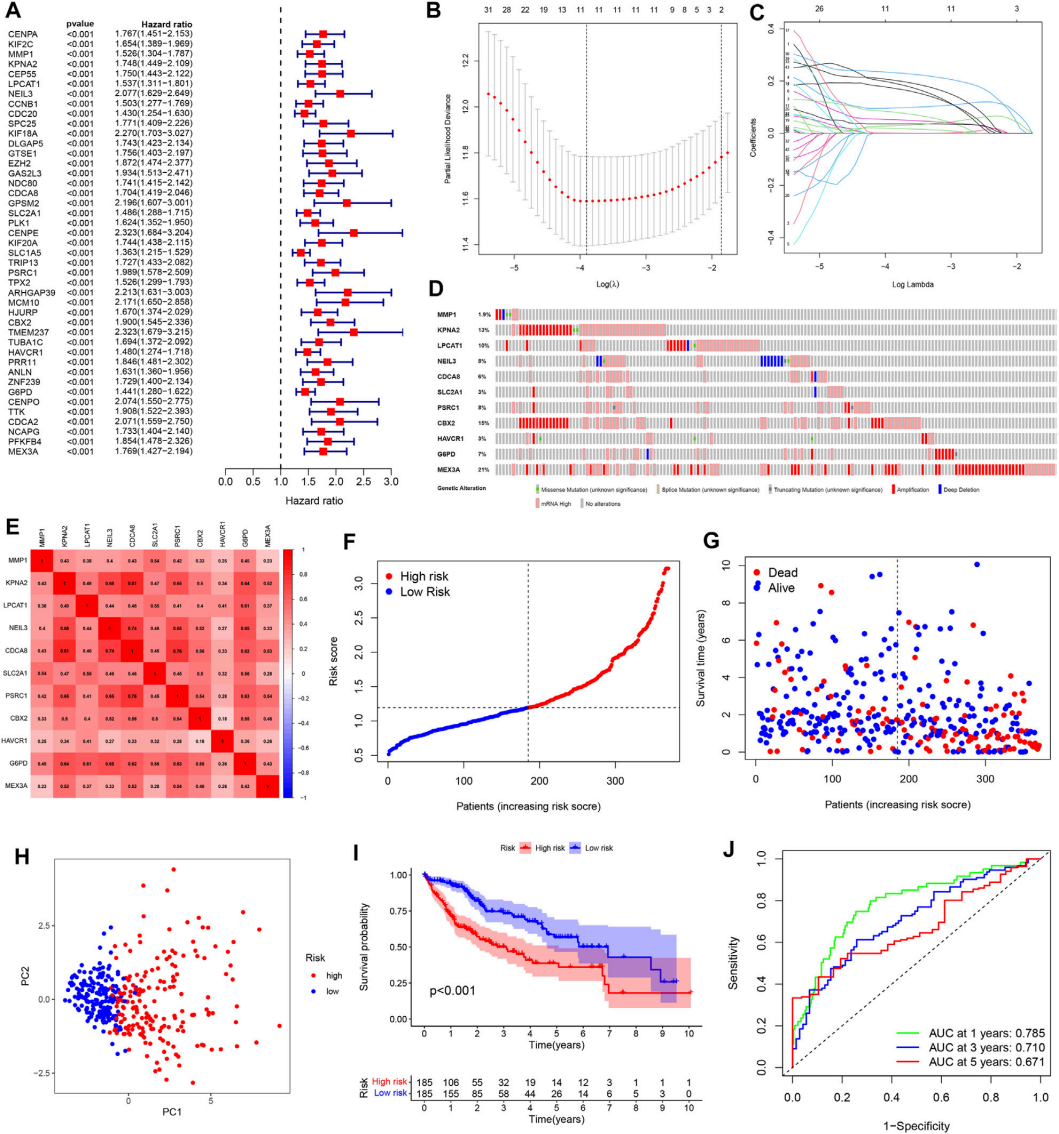
Construction of a pyroptosis risk signature in the TCGA cohort. **(A)** Univariate Cox regression analysis to find prognosis-related genes. **(B)** The Cross-Validation fit curve calculated by lasso regression method. **(C)** LASSO coefficient profiles of 11 potential prognostic genes. **(D)** Genetic alterations of 11 prognostic genes in HCC by cBioPortal database. **(E)** Spearman correlation analysis of eleven genes in the TCGA cohort. **(F)** The distribution and median value of the risk scores. **(G)** Patient survival status distribution in the high- and low-risk groups. **(H)** PCA plot analysis. **(I)** Kaplan-Meier overall survival curves for patients assigned to high- and low-risk groups based on the risk score. **(J)** ROC curve showing the prognostic value of pyroptosis risk scores on the 1-, 3-, and 5 years survival rate.

Considering the two-sided effect of pyroptosis on tumors, we further explored the prognostic value of pyroptosis risk signature. Univariate Cox regression analysis was performed to obtain 43 prognosis-related genes in the TCGA cohort, and the results showed that all genes were high-risk genes in the HCC (HR > 1, Figure 4A). To shrink the range of candidate genes for building prognostic model, a Lasso Cox regression was applied to the training cohort. Eleven genes including MMP1, KPNA2, LPCAT1, NEIL3, CDCA8, SLC2A1, PSRC1, CBX2, HAVCR1, G6PD, MEX3A, and their coefficients were eventually maintained, and the optimum *λ* value was determined via the minimum parameter (Figures 4B,C). The formula for calculating the risk score is determined as follows:

$$\begin{array}{l} \text{Risk}\;\text{Score}=\left(0.082\times \text{MMP}1\right)+\left(0.096\times \text{KPNA}2\right)\\ +\left(0.052\times \text{LPCAT}1\right)+\left(0.173\times \text{NEIL}3\right)+\left(0.001\times \text{CDCA}8\right)\\ +\left(0.019\times \text{SLC}2\text{A}1\right)+\left(0.036\times \text{PSRC}1\right)+\left(0.232\times \text{CBX}2\right)\\ +\left(0.204\times \text{HAVCR}1\right)+\left(0.028\times \text{G}6\text{PD}\right)+\left(0.193\times \text{MEX}3\text{A}\right). \end{array}$$

Moreover, we evaluated the genetic alterations of 11 genes in the TCGA database through the cBioPortal website. The results showed that the mutation frequencies of genes including MEX3A, CBX2, KPNA2, and LPCAT1 were 21%, 15%, 13%, and 10% respectively, with amplification being the most common alteration feature (Figure 4D). Additionally, there was a significant correlation between all 11 genes (Figure 4E). Then, we divided the patients into high- and low-risk groups based on the median risk score. Meanwhile, our study suggested that risk score was increased accompanying higher patient risk level and patients in the high-risk group had higher mortality and shorter survival times (Figures 4F,G). PCA analysis reduces the dimensionality of multivariate data and thus visualizes it graphically. Our results showed that patients in different risk groups were divided into two clusters following PCA analysis (Figure 4H). Besides, Kaplan-Meier analysis indicated that patients with high-risk score were significantly associated with poor prognosis (Figure 4I). The received operating characteristic (ROC) curve was performed to assess the accuracy and feasibility of pyroptosis risk signature to predict survival, and the results revealed that the area under the ROC curve (AUC) was 0.785 at 1 year, 0.710 at 3 years, and 0.671 at 5 years, respectively, displaying a favorable predictive value (Figure 4J).

### Validation of a Pyroptosis Risk Signature in an External Cohort

**FIGURE 5:**
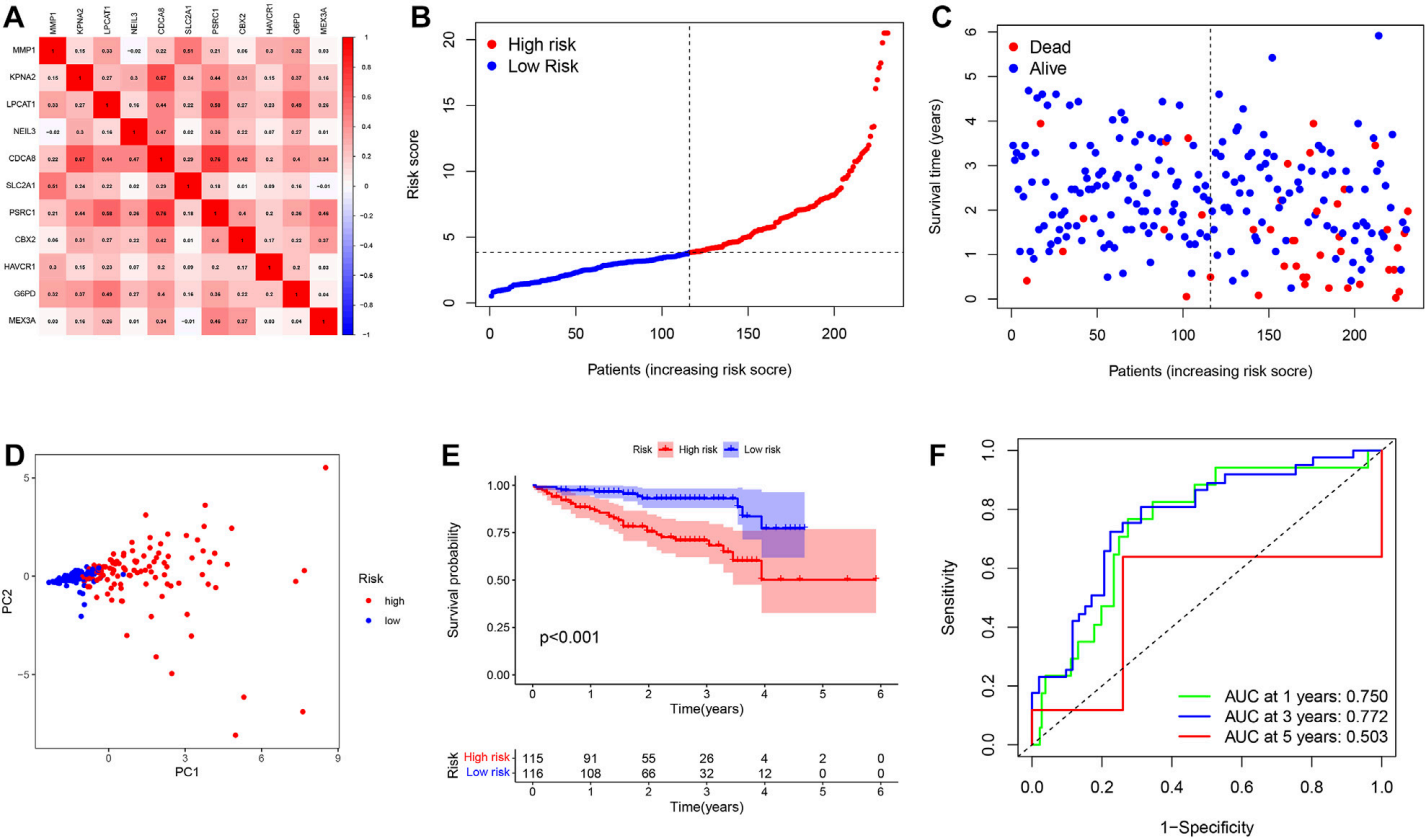
Validation of a pyroptosis risk signature in the ICGC cohort. **(A)** Spearman correlation analysis of eleven genes in the ICGC cohort. **(B)** The distribution and median value of the risk scores. **(C)** Patient survival status distribution in the high- and low-risk groups. **(D)** PCA plot analysis. **(E)** Kaplan-Meier overall survival curves for patients assigned to high- and low-risk groups based on the risk score. **(F)** ROC curve showing the prognostic value of pyroptosis risk scores on the 1-, 3-, and 5 years survival rate.

To better verify the predictive power of our risk signature, 231 patients with HCC from the ICGC database were used to create a validation cohort. As shown in Figure 5A, 11 genes were also found to be well correlated with each other in the ICGC database. Then, these cases were classified into low- and high-risk groups (Figure 5B). As with the training group, the number of deaths in the high-risk group was significantly higher compared to the low-risk group (Figure 5C). The PCA analyses demonstrated discernible dimensions between the two groups (Figure 5D). Consistently, Kaplan-Meier analysis showed that patients in the high-risk group had significantly worse survival times than those in the low-risk group (Figure 5E). In addition, the AUCs of 1, 3, and 5 year clinical outcomes were separately 0.750, 0.772, and 0.503, suggesting a good predictive efficacy (Figure 5F). It should be noted that the lack of data on patients with survival beyond 5 years in the low-risk group resulted in the AUCs at 5 years close to 0.5. Collectively, the results obtained from the validation cohort presented a satisfactory performance for the predictive capability of the risk signature.

### Independent Prognostic Value of the Pyroptosis Risk Signature

**FIGURE 6:**
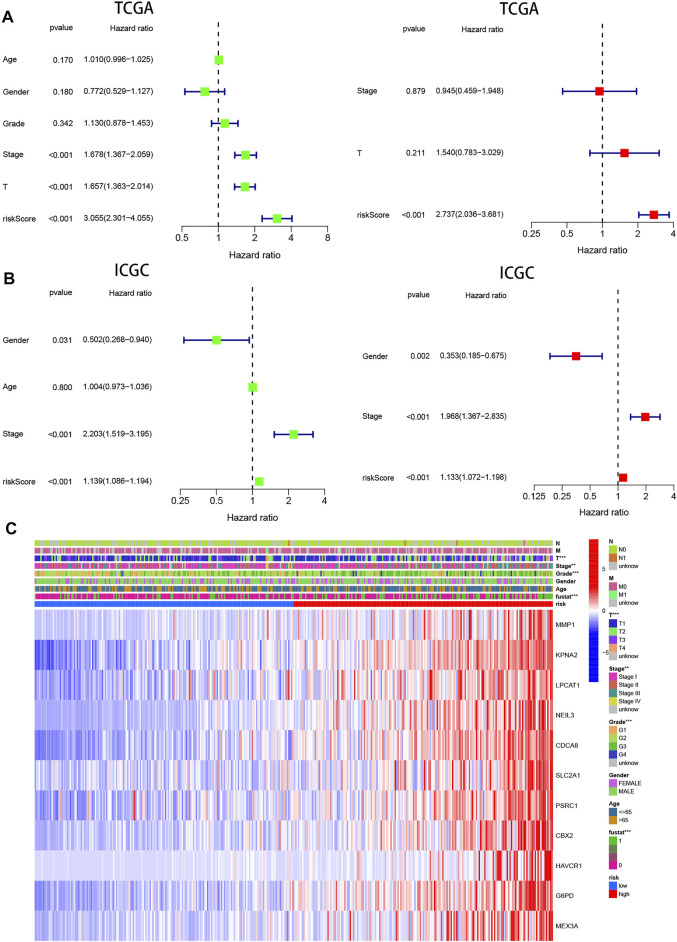
Prognostic value of the pyroptosis risk signature in HCC. **(A,B)** Univariate and multivariate Cox analyses evaluating the independent prognostic value of the pyroptosis signature in terms of OS in HCC patients in TCGA and ICGC cohorts. **(C)** Heatmap showing the relationship between clinicopathological characteristics and different risk groups. **p < 0.01, and ***p < 0.001.

Univariate and multivariable cox regression analyses were applied to analyze whether risk score could be used as an independent prognostic factor to predict prognosis. Univariate cox analysis revealed that high-risk score was markedly associated with poor prognosis (*p* < 0.001, HR = 3.055, 95 %CI: 2.301–4.055). Other variables associated with worse prognosis consisted of tumor stage and T stage. Multivariable cox demonstrated that higher risk score was independently correlated with poorer survival, indicating that it could be served as an independent prognostic factor for HCC (*p* < 0.001, HR = 2.737, 95 %CI: 2.036–3.681) (Figure 6A). These results were validated *via* the ICGC cohort, which completely echoed the above results (*p* < 0.001, HR = 1.133, 95 %CI: 1.072–1.198) (Figure 6B).

In addition, 11 gene expression profiles and clinicopathologic features from the TCGA cohort were presented in the heatmap Figure 6C and Table 1, and we found that the expression of 11 genes was significantly higher in the high-risk group and that tumor stage, grade, and patient survival status showed significant differences between the high- and low-risk groups (*p* < 0.01).

**TABLE 1:** Detailed distribution of the number of patients with different clinicopathological characteristics in low- and high-risk groups.

Covariates	Cluster	Total	Low-risk	High-risk	P value
fustat	alive	240 (64.86%)	136 (73.51%)	104 (56.22%)	**7.00E-04**
fustat	dead	130 (35.14%)	49 (26.49%)	81 (43.78%)	
Age	≤65	232 (62.7%)	110 (59.46%)	122 (65.95%)	0.237
Age	>65	138 (37.3%)	75 (40.54%)	63 (34.05%)	
Gender	FEMALE	121 (32.7%)	55 (29.73%)	66 (35.68%)	0.2678
Gender	MALE	249 (67.3%)	130 (70.27%)	119 (64.32%)	
Grade	G1	55 (14.86%)	41 (22.16%)	14 (7.57%)	**0**
Grade	G2	177 (47.84%)	97 (52.43%)	80 (43.24%)	
Grade	G3	121 (32.7%)	44 (23.78%)	77 (41.62%)	
Grade	G4	12 (3.24%)	1 (0.54%)	11 (5.95%)	
Grade	unknow	5 (1.35%)	2 (1.08%)	3 (1.62%)	
Stage	Stage I	171 (46.22%)	101 (54.59%)	70 (37.84%)	**0.0035**
Stage	Stage II	85 (22.97%)	35 (18.92%)	50 (27.03%)	
Stage	Stage III	85 (22.97%)	32 (17.3%)	53 (28.65%)	
Stage	Stage IV	5 (1.35%)	3 (1.62%)	2 (1.08%)	
Stage	unknow	24 (6.49%)	14 (7.57%)	10 (5.41%)	
T	T1	181 (48.92%)	109 (58.92%)	72 (38.92%)	**8.00E-04**
T	T2	93 (25.14%)	36 (19.46%)	57 (30.81%)	
T	T3	80 (21.62%)	33 (17.84%)	47 (25.41%)	
T	T4	13 (3.51%)	4 (2.16%)	9 (4.86%)	
T	unknow	3 (0.81%)	3 (1.62%)	0 (0%)	
M	M0	266 (71.89%)	130 (70.27%)	136 (73.51%)	1
M	M1	4 (1.08%)	2 (1.08%)	2 (1.08%)	
M	unknow	100 (27.03%)	53 (28.65%)	47 (25.41%)	
N	N0	252 (68.11%)	122 (65.95%)	130 (70.27%)	0.6704
N	N1	4 (1.08%)	1 (0.54%)	3 (1.62%)	
N	unknow	114 (30.81%)	62 (33.51%)	52 (28.11%)	

Bold values represent statistically significant.

### Construction and Validation of the Prognostic Nomogram

**FIGURE 7:**
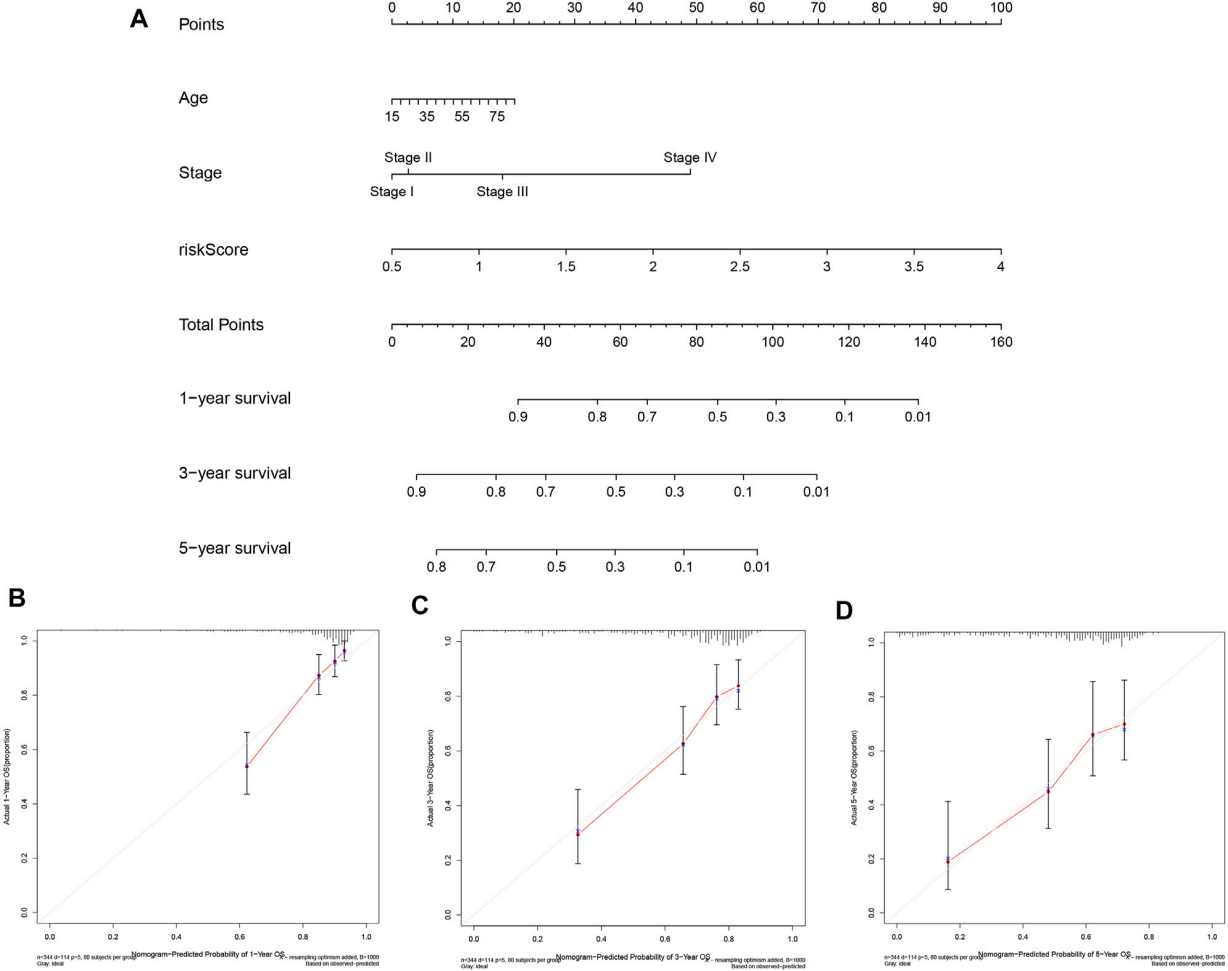
Construction and Validation of the Prognostic Nomogram. **(A)** Prognostic nomogram for predicting OS probability of patients at 1, 3, 5 years. **(B–D)** Calibration curves of nomograms for predicting 1, 3, and 5 year survival probability in TCGA cohort.

To predict the prognosis of patients more intuitively, a nomogram was created to predict the probability of OS at 1, 3, 5 years (Figure 7A). Variables including age, stage, and risk score were enrolled in the nomogram, and the total score obtained by summing up all of the scores corresponding to each variable was used to calculate the survival probability of each individual. In addition, as seen in Figures 7B–D, the calibration plots indicated a favorable agreement of the prognostic nomogram between the actual and predicted probabilities. Overall, our data suggested that the nomogram had high confidence in predicting patient survival at 1, 3, 5 years and hold promise for improved clinical application.

### GSEA Identifies Pyroptosis-Related Signaling Pathways

**FIGURE 8:**
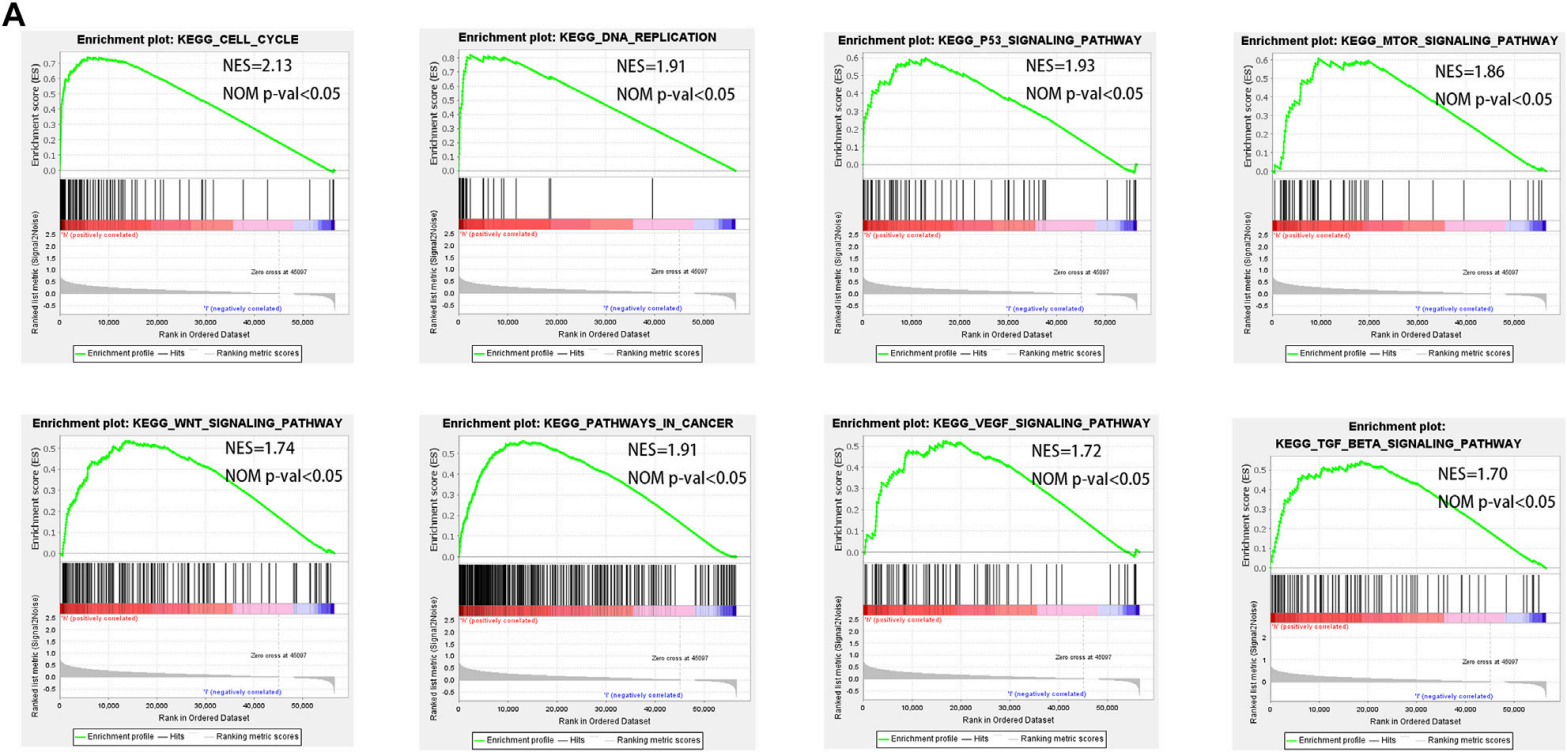
GSEA enrichment between low- and high-risk groups. **(A)** GSEA analysis showing that genes were enriched for the characteristics of malignant tumor in the high-risk group.

To understand the molecular mechanisms that may be involved in HCC and find new potential therapeutic targets, we applied GSEA to compare the high and low-risk groups. The signaling pathways enriched in the high-risk group were associated with processes that promote tumor development, such as cell cycle, DNA replication, p53 signaling pathway, MTOR signaling pathway, pathways in cancer, VEGF signaling pathway, TGF-β signaling pathway, and WNT signaling pathway (Figure 8A).

### Pyroptosis-Related Risk Signature Was Significantly Associated With Tumor Immune Microenvironment

**FIGURE 9:**
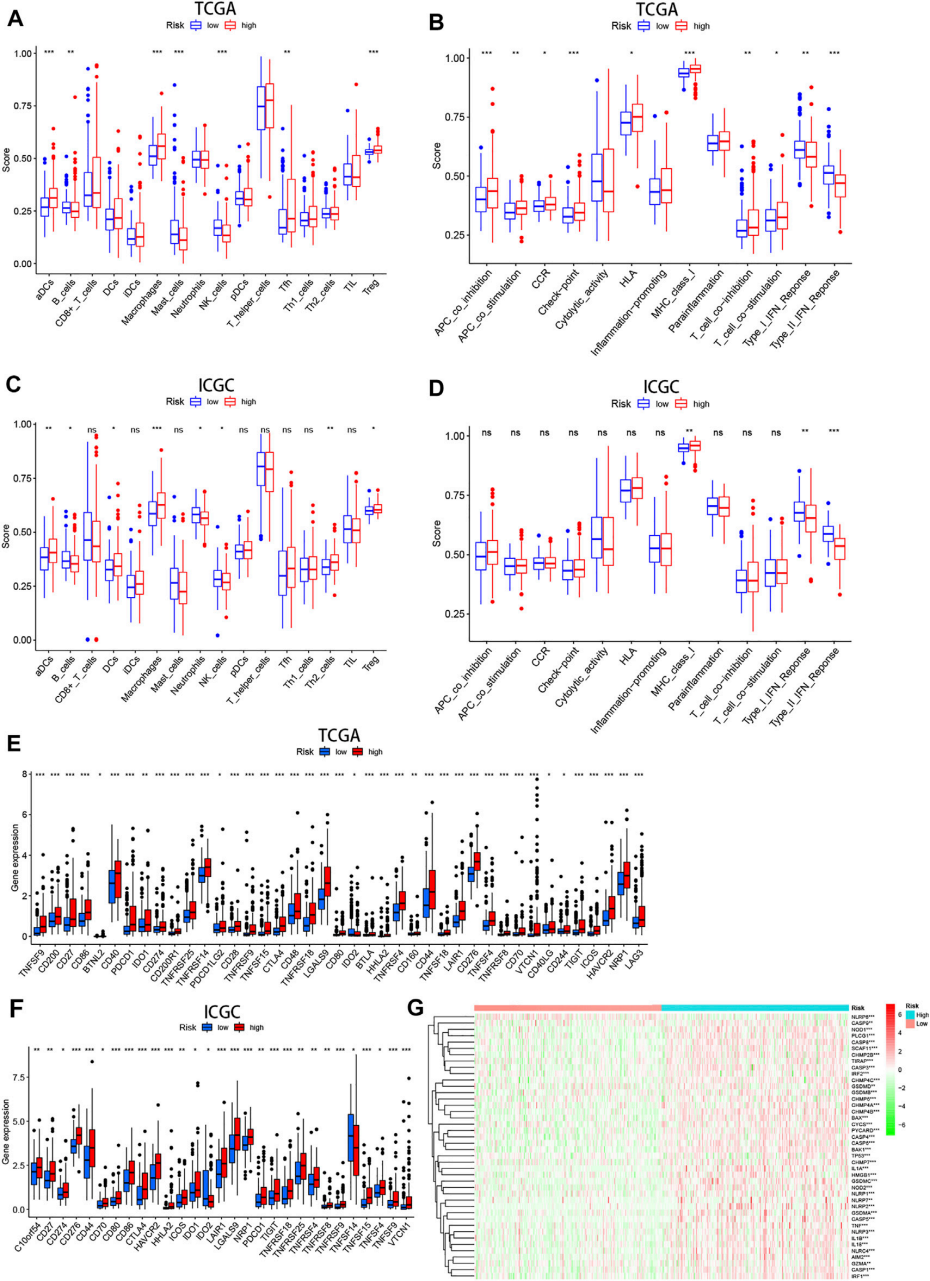
The immune status difference between high- and low-risk HCC patients. **(A–D)** Comparison of immune cell abundance and immune pathway activation in high- and low-risk groups in the TCGA and ICGC cohort. **(E,F)** Immune checkpoint expression in high- and low-risk groups in the TCGA and ICGC cohort. **(G)** Heatmap showing expression of the pyroptosis-related genes in the high- and low-risk groups.

To further explore the effect of pyroptosis signature on the immune microenvironment, ssGSEA was applied to analyze the level of immune cell infiltration and activation of immune-related functional pathways in high- and low-risk groups. We found that patients with high-risk score had significantly higher proportions of immune cells including aDCs, Macrophages, Tfh, Treg, but significantly lower proportions of B cells, Mast cells, NK cells (Figure 9A). In addition, there were statistically significant differences in the score of immune-related functions, except for the Cytolytic activity, Inflammation promoting, and Parainflammation in the high- and low-risk groups (Figure 9B). Regarding ssGSEA analysis in the ICGC cohort, the results for the level of immune cell infiltration were generally consistent with the TCGA cohort (Figure 9C), but only three immune-related functions (e.g., MHC class I, Type-I IFN response, and Type-II IFN response) showed significant differences (Figure 9D).

On the basis of previous study that immune checkpoints play an essential role in tumor immune escape, we examined the expression of these molecules between high- and low-risk groups. We found that patients with high-risk score were characterized by high expression of most immune checkpoints (i.e., PDCD1, CTLA-4, HAVCR2, LAG-3, and et al.) (Figures 9E,F). These results suggest that patients with high risk are more likely to form an immunosuppressive tumor microenvironment by upregulating the expression of these molecules. Moreover, the heatmap showed that pyroptosis-related genes were generally upregulated in HCC patients with high-risk score (Figure 9G).

### High Risk Score Tended to Chemotherapy Resistance

**FIGURE 10:**
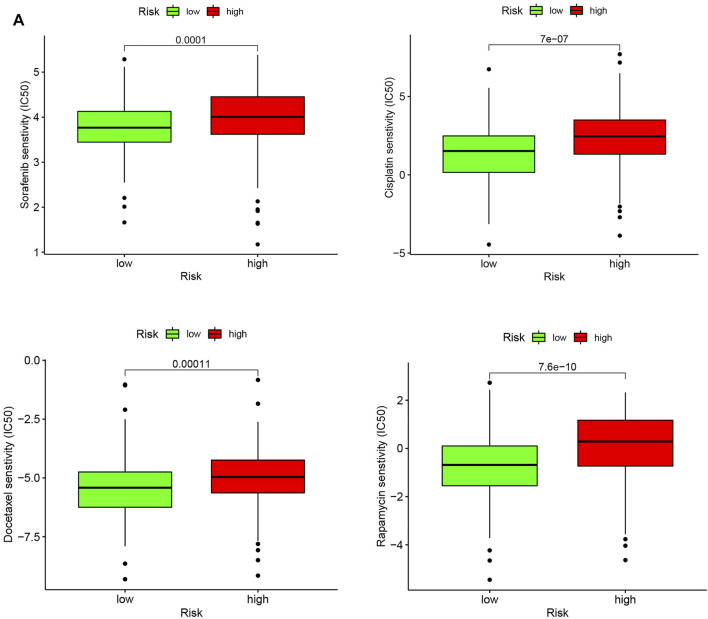
Chemotherapeutic response in the high-risk and low-risk groups. **(A)** Box plot visualizing the IC50 of Sorafenib, Cisplatin, Docetaxel, and Rapamycin between low- and high-risk HCC patients.

Chemotherapy resistance is a common phenomenon in the treatment of advanced tumors, and it is also an insurmountable difficulty at present. In the present study, we investigated the IC50 values of four common chemotherapy agents for HCC including Sorafenib, Cisplatin, Docetaxel, and Rapamycin in high- and low-risk groups. Our results showed that the IC50 values for the four chemotherapy agents were significantly higher in the high-risk group, indicating that patients with high-risk score were more prone to develop chemoresistance (Figure 10A).

## Discussion

HCC remains one of the most lethal malignancies, with the second-highest mortality rate of all cancers worldwide (Mattiuzzi and Lippi, 2020). An increasing number of studies have confirmed the important role of pyroptosis in tumors, and the findings of the relationship between pyroptosis and tumors are not entirely consistent, indicating the heterogeneity of tumors and the complexity of the immune microenvironment. Pyroptosis can not only impair the progression of the tumor but also create a microenvironment suitable for tumor cell growth and thus contribute to tumor development (Xia et al., 2019). In recent years, many models have been constructed based on data mining of gene expression profiles and clinical outcomes of HCC(Liu et al., 2020b; Hong et al., 2020). However, the perception about the diagnostic and prognostic value of pyroptosis for HCC is still insufficient.

In the present study, we performed consensus clustering to identify two clusters based on 42 pyroptosis-related DEGs, which showed significant survival differences in clusters one and 2. The DEGs between the two clusters were then explored, and the results showed enrichment in terms of functions associated with immune response. To further investigate the prognostic value of pyroptosis on HCC, DEGs were analyzed using univariate Cox and LASSO Cox regression analysis to construct an 11-gene risk signature. GSEA analysis revealed that patients in the high-risk group were associated with activation of oncogenic pathways. When exploring the state of the immune microenvironment, there was a significant difference in the level of immune cell infiltration and immune-related pathway activation in patients with high and low-risk score, and the expression of immune checkpoints was significantly higher in the high-risk group. Drug sensitivity analysis indicated worse chemotherapy outcomes in high-risk groups. The results of this study highlight the potential research value of pyroptosis in HCC.

Here, our pyroptosis risk signature was constructed from 11 risk molecules, including MMP1, KPNA2, LPCAT1, NEIL3, CDCA8, SLC2A1, PSRC1, CBX2, HAVCR1, G6PD, MEX3A, of which KPNA2, LPCAT1, CBX2, MEX3A had higher mutation frequencies in patients with HCC. Previous reports indicate that KPNA2 contributed to the inflammatory processes in tumors (Cai et al., 2016) and was also involved in the carcinogenesis of various malignancies such as melanoma (Yang et al., 2020b), HCC (Zan et al., 2019), colon cancer (Takada et al., 2016), and so forth, and high expression of KPNA2 was associated with poor outcomes of patients. Mao et al. (2019) identified that interfering with the expression of CBX2 inhibits HCC cell proliferation and increases apoptosis. Liu et al. (2020a) emphasized the important research value of the miR-205-LPCAT1 axis in regulating the progression of various tumors. MEX3A was overexpressed in HCC tissue and was also identified as an independent prognostic factor for HCC patients (Yang et al., 2020a). MMP1, a member of the zinc-dependent endopeptidase family, has been proved to be associated with proliferation and metastasis in various cancers (Kessenbrock et al., 2010). Zhou et al. (2017) proposed that NEIL3 maintains genome stability during the S/G2 phase by targeting repair of oxidative damage at telomeres. Interestingly, recent studies indicate that NEIL3 contributes to repairing oxidative telomere damage at mitosis, which is crucial for fighting senescence in HCC cells (Zhao et al., 2021). Knockdown of CDCA8 inhibits HCC cell progression by restoring ATF3 tumor suppressor and inactivating AKT/beta-Catenin signaling (Jeon et al., 2021). SLC2A1 was the gene encoding glucose transporter 1 (Glut-1). GLUT1/SLC2A1, a uniporter that was expressed by various carcinomas, may participate in malignant neoplasm glycometabolism and was associated with the prognosis of gliomas patients (Komaki et al., 2019). Overexpression of PSRC1 promotes the expression of genes related to cell proliferation (Meroni et al., 2021). Ye et al. (2018). identified a potential mechanism of TIM-1(HAVCR1)+Breg cell-mediated immune evasion in HCC. G6PD was also up-regulated in HCC as well as promoting cell invasion and migration (Lu et al., 2018).

It is generally accepted that there is a close interaction between tumors and the complex immune system, and various cancer immunotherapies have been designed to identify and eliminate tumor cells. Natural killer (NK) cells are an essential component of anti-tumor immunity, which can not only directly kill tumor cells, but also affect the anti-tumor behavior of other immune cells (Yuen et al., 2016). Granzyme B in NK cells possessed the same cleavage site as caspase-3, which can cleave GSDME to induce pyroptosis (Zhang et al., 2020). Previous studies have observed that GSDME-induced pyroptosis to suppress tumors was disappeared in mice lacking NK cells and CD8^+^ T cells, suggesting that this inhibitory effect is reliable on these two immune effector cells in the immune system (Zhang et al., 2020). Our results show that the infiltration of NK cells in the high-risk group was significantly reduced. Although CD8^+^ T cells were not significantly different, the absence of NK cells may affect tumor cells being induced to pyroptosis.

Besides, increasing studies observed that tumor-associated macrophages were associated with tumor-promoting inflammation and may favor tumor initiation and progression (Mantovani et al., 2017; Ngambenjawong et al., 2017). Meanwhile, high-level intratumoral Tregs designed a generalized immunosuppressive tumor microenvironment and protected tumor cells from the host’s immune surveillance (Nishikawa and Koyama, 2021). Previous study proposed that the presence of B cells in tumors was associated with a better prognosis for patients receiving immunotherapy, and they speculate that B cells may support CD8+T cells to effectively fight tumor cells (Cabrita et al., 2020; Petitprez et al., 2020). Our data illustrated that the infiltration levels of macrophages and regulatory T cells (Tregs) were upregulated for HCC patients with high-risk score, while B cells were downregulated.

Notably, our study found that aDCs were significantly higher in the high-risk group. It has been shown that dendritic cells are the most important cell type for initiating cancer T-cell responses (Hildner et al., 2008; Binnewies et al., 2019), which is a particular advantage for patients with high-risk score. In addition, we found that the type I and II IFN response were decreased in the high-risk group, which was validated in the cohort. Type I interferons (Type I IFNs) are involved in the process of cancer immunoediting, which can not only inhibit the recruitment and activation of Tregs (Hashimoto et al., 2014; Hirata et al., 2019), but also the depletion of Type I IFNs affects the intensity of NK cells in anti-tumor immune responses (Rautela et al., 2015). Type II IFN (IFN*γ*) treatment can cause cell cycle arrest and inhibit the growth of pancreatic cancer cells by triggering caspase-1- and IRF1-dependent apoptosis (Detjen et al., 2001). These findings suggested that type I and II IFN response may be involved in the pyroptosis-mediated immunosuppression.

Apart from the complex role of pyroptosis in tumors, it has a double-edged sword-like effect in the tumor immune microenvironment. Reck et al. (2019) reported that PD-L1 inhibitor combined with radiotherapy or chemotherapy triggers pyroptosis-induced inflammation within the tumor microenvironment to kill tumor cells. Additionally, Chui et al. (2019) proposed that DPP8/9 inhibitors could cleave NLRP1b to release the C-terminus, thereby triggering caspase-1-induced pyroptosis. Interestingly, our data illustrated that most of the immune checkpoints (PD-L1, PDCD1, TIM3, CTLA4, LAG3, and et al.) were upregulated at the high-risk group, as were the pyroptosis related-genes, indicating that patients with high-risk score may be able to achieve desired therapeutic outcomes when treated with immune checkpoint inhibitors.

Moreover, GSEA analysis indicated that a variety of pathways (e.g., cell cycle, TGF-*β* signaling pathway, pathways in cancer, and so on) involving the development and progression of tumors were activated in the high-risk group. It is widely accepted that dysregulation of the cell cycle was considered an important marker of tumors (Dominguez-Brauer et al., 2015). Haque et al. (Haque and Morris, 2017) found that TGF-*β* disturbed the stabilization of the immune system by inhibiting the activation of NK cells and reducing cytokine production. Meanwhile, the results of drug prediction showed that high-risk patients were less sensitive to HCC chemotherapy drugs, including Sorafenib and others. Sorafenib is indicated as a first-line treatment option for patients with unresectable or metastatic advanced HCC. Therefore, future research should be directed at exploring the mechanisms between pyroptosis and drug resistance.

Of course, some limitations of this study have to be considered. First, we need more multicenter and prospective clinical cohorts to validate the predictive value of 11-gene pyroptosis signature for HCC survival in the future. Second, the activated signaling pathways in the high-risk group should be validated *in vivo* and *in vitro* experiments. Additionally, the relationship between pyroptosis signature and the overall intensity of immune responses within the HCC microenvironment should be further investigated.

## Conclusion

In this study, we successfully established and validated a 11-gene risk signature that could serve as an independent prognostic factor for HCC patients. High-risk patients have a worse prognosis and also multiple carcinogenesis-related pathways were activated in high-risk group. Analysis of the tumor immune microenvironment revealed that some immune effector cell infiltration was reduced in the high-risk group, while immunosuppressive cell infiltration was increased, and patients with high-risk score were more prone to receive treatment with immune checkpoint inhibitors. Because of the large variation between patients, our model can guide clinicians to provide support for individualized treatment of patients. Overall, the potential of pyroptosis for oncology treatment will become a promising and noteworthy area in cancer research.

## Data Availability

The original contributions presented in the study are included in the article/Supplementary Material, further inquiries can be directed to the corresponding authors.

## Abbreviations

DEGs, differentially expressed genes; GO, Gene Ontology; HCC, hepatocellular carcinoma; KEGG, Kyoto Encyclopedia of Genes and Genomes; NK, natural killer; OS, overall survival; PPI, protein-protein interaction network; ROC, received operating characteristic; ssGSEA, single-sample gene set enrichment analysis.
