# Supplementary material for: Development and Validation of a Pyroptosis-Related Signature for Predicting Prognosis in Hepatocellular Carcinoma
Source: Front Genet. 2022 Jan 24;13:801419. doi: 10.3389/fgene.2022.801419 (PMC8818951; doi:10.3389/fgene.2022.801419)
Supplement: Supplementary file 1 [file Table1.docx]

BAK1

BAX

CASP1

CASP3

CASP4

CASP5

CHMP2A

CHMP2B

CHMP3

CHMP4A

CHMP4B

CHMP4C

CHMP6

CHMP7

CYCS

ELANE

GSDMD

GSDME

GZMB

HMGB1

IL18

IL1A

IL1B

IRF1

IRF2

TP53

TP63

AIM2

CASP6

CASP8

CASP9

GPX4

GSDMA

GSDMB

GSDMC

IL6

NLRC4

NLRP1

NLRP2

NLRP3

NLRP6

NLRP7

NOD1

NOD2

PJVK

PLCG1

PRKACA

PYCARD

SCAF11

TIRAP

TNF

GZMA
